# Animal, Plant, Collagen and Blended Dietary Proteins: Effects on Musculoskeletal Outcomes

**DOI:** 10.3390/nu12092670

**Published:** 2020-09-01

**Authors:** Colleen S Deane, Joseph J Bass, Hannah Crossland, Bethan E Phillips, Philip J Atherton

**Affiliations:** 1 Department of Sport and Health Sciences, College of Life and Environmental Sciences, University of Exeter, Exeter EX1 2LU, UK; c.s.deane@exeter.ac.uk; 2 Living Systems Institute, University of Exeter, Stocker Road, Exeter EX4 4QD, UK; 3 MRC versus Arthritis Centre for Musculoskeletal Ageing Research and NIHR Nottingham Biomedical Research Centre, University of Nottingham, Royal Derby Hospital Centre, Derby DE22 3DT, UK; Joseph.Bass2@nottingham.ac.uk (J.J.B.); Hannah.Crossland1@nottingham.ac.uk (H.C.); Beth.Phillips@nottingham.ac.uk (B.E.P.)

**Keywords:** animal-derived protein, plant-derived protein, collagen-derived protein, protein blends, skeletal muscle, bone, ageing, exercise, energy restriction

## Abstract

Dietary protein is critical for the maintenance of musculoskeletal health, where appropriate intake (i.e., source, dose, timing) can mitigate declines in muscle and bone mass and/or function. Animal-derived protein is a potent anabolic source due to rapid digestion and absorption kinetics stimulating robust increases in muscle protein synthesis and promoting bone accretion and maintenance. However, global concerns surrounding environmental sustainability has led to an increasing interest in plant- and collagen-derived protein as alternative or adjunct dietary sources. This is despite the lower anabolic profile of plant and collagen protein due to the inferior essential amino acid profile (e.g., lower leucine content) and subordinate digestibility (versus animal). This review evaluates the efficacy of animal-, plant- and collagen-derived proteins in isolation, and as protein blends, for augmenting muscle and bone metabolism and health in the context of ageing, exercise and energy restriction.

## 1. Skeletal Muscle, Bone, Protein Sources and the Notion of Protein “Quality”

Dietary protein can attenuate skeletal muscle and bone decline during ageing [1,2] and energy restriction [3,4] and can potentiate exercise-induced increases in muscle and bone mass and/or function [5]. However, concerns regarding the sustainability of animal-derived proteins [6] has led to an emerging interest in the efficacy of plant-derived and other (e.g., collagen-derived, blended) protein sources for maintaining/optimising musculoskeletal health, which is currently a hotbed of research.

### 1.1. Definition of Animal, Plant, Collagen and Blended Dietary Protein Sources

From the outset, it is important that we define what is meant by animal, plant, collagen and blended dietary proteins herein, to provide clarity and prevent misinterpretation. Animal-derived refers to proteins *directly* originating from animal sources such as meat, fish, poultry, eggs and dairy (and the constituents whey and casein protein) [7], which are also regarded as “complete” proteins (i.e., they provide sufficient amounts of all essential amino acids (EAA) to meet human requirements) [8]. Plant-derived refers to proteins obtained from plant sources (e.g., wheat, soy) [9] and collagen-derived refers to proteins derived from gelatin and/or collagen hydrolysates [8,10]. Notably, gelatin/collagen hydrolysates-derived proteins do originate from animal sources (e.g., bone, pigskin, fish skin [10]), however, they are not regarded as “complete” proteins, hence our rationale for distinguishing them from animal-derived protein sources for the purpose of this review. Finally, blended protein sources refer to different sources/types of protein combined together to form one nutritional load.

### 1.2. Muscle and Bone Protein Turnover

In the simplest form, dietary protein can modulate muscle and bone health via the regulation of muscle protein turnover [4,11] and bone matrix turnover and remodelling [12,13], respectively. In regards to muscle, the global maintenance of skeletal muscle mass is governed by the dynamic equilibrium between muscle protein synthesis (MPS) and muscle protein breakdown (MPB), where a positive net muscle protein balance (i.e., MPS exceeds MPB) results in muscle growth and a negative net muscle protein balance (i.e., MPB exceeds MPS) results in muscle loss [11]. Dietary protein provides a critical source of amino acids (AA), which act as protein synthetic precursors and modulate anabolic signalling activity, stimulating robust increases in MPS [9]. Further, protein-derived AA can attenuate MPB, which is entirely attributable to insulin, contributing to a positive net protein balance [14]. In regard to bone, the cross-linking of collagen molecules involves the post-translation modification of AA, therein requiring dietary-derived AA since many of the collagen fragments released during breakdown cannot be re-utilised for bone matrix formation [15]. However, dietary protein can also increase urinary calcium excretion, therein possibly increasing the risk of fractures or osteoporosis, which has led to some controversy surrounding the efficacy of dietary protein for bone health [15].

### 1.3. Dietary Protein Requirements

The Recommended Daily Allowance (RDA) of dietary protein for adults (including older adults), is currently 0.8 g/kg of high-quality protein per day [16], which is based on the minimum dietary protein required to achieve nitrogen balance, thus maintaining body protein mass [17]. However, short-comings associated with the nitrogen-balance technique have led to criticism and a call for the protein RDA to be increased [17,18], particularly in the context of ageing, exercise, energy restriction and disease, where protein anabolism and nitrogen excretion are affected [17,18,19]. Further, the current RDA does not take into account the source of protein (i.e., animal, plant, collagen, blended), which is an important consideration since there are known geographical differences in regard to the source of protein intake. To highlight, western diets contain a higher proportion of total protein intake from animal-derived compared to plant-derived protein sources [20,21]. Dietary analysis from the National Health and Nutrition Examination Survey 2003–2006 study [21] indicated that 65% of protein intake in US populations was from a combination of animal-derived sources, similar to the 66% reported in the more recent (also US-based) PREMIER survey [20]. In comparison, African and Asian populations’ primary source of protein is plant-derived, at 77% and 66% of consumption, respectively [7]. Moreover, in the western population, there is an apparent shift towards increased consumption of plant-derived protein (in lieu of animal), due to perceived health benefits (e.g., reduced cardiovascular mortality [22]) and environmental sustainability [6]. Reflecting this shift, while the majority of prior research investigations have focused upon characterising the effects of animal-derived proteins in relation to musculoskeletal metabolism and health, there has been a recent surge of studies investigating the influence of plant-derived (e.g., References [4,23,24,25]), collagen-derived (e.g., References [26,27,28,29]) and blended (e.g., References [30,31,32]) protein sources.

### 1.4. Protein Quality

The quality of a protein source is thought to be a central factor in its ability to provide a physiological benefit, and is dependent upon the proportion of protein-derived AA from digestion and absorption processes [7]. Protein quality is typically evaluated by the Protein Digestibility Corrected Amino Acid Score (PDCAAS); however, the more recently introduced Digestible Indispensable Amino Acid Score (DIAAS) allows for consideration of ileal AA digestibility, permitting a more accurate assessment and indication of specific rate-limiting AA [33]. Thus, PDCAAS or DIAAS values theoretically afford insight into protein requirements to maintain whole-body nitrogen and AA balance; however, crucially, neither assessment discerns systemic or tissue-specific (i.e., musculoskeletal) impacts. For example, while beef and soy protein have similar PDCAAS values (92 and 91, respectively [34]), ingestion of 4 oz of beef stimulates postprandial myofibrillar MPS responses to a greater degree than ingestion of 4 oz of isonitrogenous soy protein [35]. Although, it should be considered that this may also be due to differences in the macronutrient composition/food matrix (i.e., structure and interaction of feed components [36,37]) of beef vs. soy protein. For a summary of animal- and plant-derived DIAAS and PDCAAS scores, the readers are directed to the work of Burd et al. [37].

Therefore, the purpose of this narrative review is to consider the continually developing impacts of animal- (Section 2), plant- (Section 3) and collagen-derived (Section 4) protein sources (incorporating both acute and chronic study designs) in relation to skeletal muscle and bone metabolism and health. Where sufficient data exists, this will be considered in the context of ageing [38], exercise [39] and energy restriction [40], as conditions that negatively or positively affect musculoskeletal metabolism and health. We will also address the emerging potential of protein blends as sustainable anabolic sources for musculoskeletal health (Section 5) and finally, we will highlight future research directions for each given context (Section 6). We would like to iterate that this is a narrative review, which includes studies based on the suitability with the aforementioned criteria (i.e., animal and/or plant and/or collagen feeding in the context of ageing, exercise, energy restriction on musculoskeletal outcomes). Since we have not performed a systematic analysis, we would like to apologise to those authors who’s work we may have unintentionally omitted from this review.

## 2. Animal-Derived Proteins: Effects in Relation to Age, Exercise, Energy Restriction and Source

### 2.1. Skeletal Muscle

The importance of dietary protein for skeletal muscle maintenance is undeniable. In young healthy adults, animal-derived protein sources robustly increase acute MPS [41], which is entirely attributable to the EAA content [42]. Of the EAA, the branched chain amino acids [43], and in particular, leucine [14], provide the most potent anabolic stimulation. Further, protein-induced increases in MPS are saturable and finite, with 20–40 g of animal-derived protein [41,44,45] (or 10–20 g EAA [46]) stimulating maximal MPS, which increases ~45–60 min following oral consumption (time taken for digestion and absorption), reaching maximal stimulation (~two- to three-fold) between 1.5 and 3 h and returning to baseline ~2–3 h post-consumption [39,47]. Interestingly, MPS levels return to baseline in spite of continued muscle and plasma AA availability and elevated anabolic signalling [48], suggesting that the muscle remains refractory to dietary protein-induced MPS stimulation for a currently unknown period of time [39,47], which has been coined “muscle-full” [48].

Given the utility of animal-derived protein intake in young healthy populations, a number of large cohort studies have assessed the relationship between animal-derived protein intake and muscle health across age. For example, in a study by Alexandrov et al. [49], muscle mass estimates from 24 h urinary creatinine and analysis of food intake by questionnaires illustrated that increased intake of both total protein and animal protein were associated with increased creatinine excretion (i.e., higher muscle mass) in both young and older males and females. Similarly, data from the Framingham Offspring Study found that higher protein intake from animal sources (e.g., red meat, poultry, fish) was associated with a higher percentage muscle mass over a 9-year period in adults over the age of 50 years [1]. These findings point towards positive effects of animal protein sources for the maintenance of muscle mass across the lifespan.

With this in mind, determining the efficacy of animal-derived protein feeding for potentiating muscle health in older adults has been a key aim of several investigations. Indeed, many studies have demonstrated that dairy [50] and meat [41,51] protein sources stimulate MPS in older adults. To demonstrate, one short-term study assessing the effects of a moderate (30 g) versus large (90 g) serving of 90% lean beef on MPS in younger (~35 years) and older (~68 years) adults found that MPS similarly increased in both age groups in response to the moderate serving of protein, with no further increase seen with the larger serving in either young or older adults [41]. This data is suggestive of a ceiling effect in healthy rested individuals in response to a single serving of animal protein, in line with the aforementioned “muscle full” hypothesis [48]. Interestingly, this data (and others [51,52]) does not support the notion of “anabolic resistance”, which states that ageing muscle displays attenuated protein synthetic responses to protein feeding (and exercise) [53]. This is in disagreement with several studies that have shown anabolic resistance in response to feeding with EAA [46,54] and animal-derived protein [55]. To demonstrate, a retrospective cross-sectional study found that older adults exhibited a blunted protein synthetic response following 20 g casein protein consumption, compared to their younger counterparts [55]. This particular study pooled multiple well-controlled trials with similar study designs, thereby accruing a large volunteer pool (compared to other similar studies), and thus provides strong evidence to support the existence of anabolic resistance in ageing [9,55]. Although the mechanisms underlying anabolic resistance remain to be fully elucidated, a suggested contributor is the rate of protein digestion and AA absorption, which may impact the postprandial availability of AA for MPS [9,56], whereby compared to slowly digestible proteins, more rapidly digestible proteins result in a greater postprandial stimulation of MPS [57]. Whilst it has been shown on multiple occasions that older adults ingesting 20 g whey protein increased MPS to a greater extent than those ingesting 20 g casein protein (which has slower digestion and absorption properties compared to whey protein), leucine content was higher in whey protein, which is more likely the key anabolic driver [58,59]. Additionally, pulse feeding, which results in lower and more gradual aminoacidemia and leucinemia compared to bolus feeding, elicited equivalent net muscle anabolism in older adults (compared to bolus), suggesting that the speed of digestibility does not affect MPS [60]. The matrix and texture of the feed, which can be a consequence of food processing (e.g., mechanical processing such as mincing [57]), is another factor modulating the digestion and absorption and thus, potentially the protein synthetic response to animal-derived protein [57]. To highlight, Pennings et al. found that compared to beef steak, minced beef was more rapidly digested and absorbed, thereby stimulating a more rapid release of AA into circulation and thus enhancing postprandial net protein balance in older (~74 years) males, however, no differences in MPS were observed [61]. Further, irrespective of the coagulation mode, gelation of milk reduces AA absorption and the postprandial rise in circulating AAs [62,63]. Other considerations to obtain optimal digestion, absorption and synthetic kinetics in the context of ageing are chewing efficiency [64], cooking temperature [65] and cooking time [57]. As such, digestibility may modulate the anabolic response to protein feeding but this proposition remains contentious and requires further thorough investigation. It is, however, without doubt that the EAA profile of animal-derived proteins (e.g., higher leucine content) largely accounts for the robust anabolic responses to these proteins.

In more chronic experimental designs, one study assessed the effects of a 12-week diet with or without dairy-rich protein supplements in older adults [66]. Over this period, both groups saw negative changes in muscle strength, but a greater loss was observed in the control group, suggesting that protein intake might offset functional decline. In addition, the dairy protein group increased appendicular lean mass, indicating that a dairy-rich diet may be an effective strategy to counteract muscle loss in older adults. However, in older females habitually consuming more than the protein RDA, an additional daily protein drink containing 30 g whey protein (with reported 87% compliance) had no effect on muscle mass or function over a 2-year period [67], suggesting that the effectiveness of dietary protein may depend on the nutritional status and habitual protein intake of older individuals.

In regard to exercise × protein interactions, animal-derived protein sources can enhance the magnitude and duration of the increase in MPS in both young and older adults [47,68], therein delaying the “muscle full” set point [47]. In order to maximise the MPS response to acute resistance exercise (RE), research has focused on optimising protein feeding strategies, albeit mostly in younger adults. For example, Witard et al. [44] found that ingestion of 20 and 40 g whey protein isolate increased myofibrillar MPS above 20 g at rest and after unilateral RE in young health males, with no difference in MPS stimulation between 20 and 40 g. This data indicates that 20 g of whey protein is sufficient to stimulate maximal MPS post-exercise in the young with doses > 20 g leading to AA oxidation and ureagenesis, at least in the case of unilateral RE [44]. Indeed, it is not just animal-derived whey protein that can promote exercise × protein interactions. The slowly digested protein, casein, which elicits prolonged hyperaminoacidemia (likely due to slow gastric emptying) [69], has been shown to stimulate myofibrillar MPS and anabolic signalling 1–6 h post-RE [70].

Although 20 g whey protein appears to saturate MPS in young individuals, older adults appear to be responsive to greater protein doses in the context of exercise. For example, Yang et al. [71] found that in older males performing unilateral leg RE, whole-body leucine oxidation increased in a dose-dependent manner with increasing amounts of whey protein isolate (0, 10, 20 and 40 g), with rates of post-RE MPS enhanced with the highest two doses. Further, increasing amounts of protein (0, 57, 113 or 170 g) derived from ground beef elevated myofibrillar MPS both at rest and after acute RE to a greater extent in middle-aged males (~59 years) [72]. Importantly, in older adults, the source of animal protein can influence exercise × protein anabolic responses. For example, a study in healthy older individuals [73] demonstrated that a single bolus of high whey protein (20 g whey protein, 3 g total leucine) consumed immediately after RE resulted in a higher rate of MPS 4 h post-exercise than with an isocaloric milk protein control drink (6 g milk protein). Similarly, whey protein was found to stimulate MPS to a greater extent than casein protein when combined with RE in older (~72 years) males [58]. Thus, the amount and source of animal-derived protein should be considered when looking to optimise age-related anabolic responses to acute exercise.

Repeated post-exercise increases in MPS culminate over time (i.e., in response to resistance exercise training (RET)), leading to gains in muscle mass and strength, which may be potentiated with protein-feeding across age. Indeed, a study comparing young and older males found that whey protein (26.2 g AA per serving) ingestion during 12 weeks RET increased mechanistic target of rapamycin (mTOR), a “master regulator” of muscle growth, both before and after RET in younger males (compared to exercise combined with placebo) [74]. However, in older males, there was an increase in whey protein plus exercise-induced mTOR protein phosphorylation before RET, but this was diminished after, perhaps suggestive of an effect of ageing on exercise and animal-protein interactions [74]. When assessing muscle mass and functional outcomes in mobility-limited older adults completing 6 months of progressive high-intensity RET, consuming 40 g whey protein daily had no greater effect on lean mass or strength than the isocaloric (but not isoproteic) control [75]. In contrast, a recent study by Kang et al. [76] reported that following daily whey protein (32.4 g) supplementation in frail older adults undergoing 12 weeks of RET, grip strength, chair-to-stand time and gait speed improved to a greater extent in the whey protein supplementation group than in the RET only group. This data suggests that animal-derived protein can positively influence muscle function. Whilst there are conflicting reports (as outlined above), a meta-analysis of 22 studies (6 of which included older adults) concluded that animal protein feeding potentiates muscle mass and function gains during RET across age [5]. Collectively, these reports indicate that animal protein supplementation when combined with exercise training may promote muscle mass and function, however, in older adults, the outcomes may depend on the protein dose, the duration of supplementation and/or the characteristics of the volunteers.

Hospitalisation, illness and/or advancing age can lead to a reduced appetite and a subsequent reduction in nutrient intake, leading to a hypoenergetic state and muscle loss [77]. This situation also presents during purposeful weight loss in the form of a reduced calorie diet, hence the need for optimal nutritional interventions that aim to preserve muscle mass and function in the face of energy restriction. In a recent study by Hector et al. [4], males and females aged between 35 and 65 years consumed either whey protein (27 g) or soy protein (26 g) supplements during a 14-day weight loss diet. Postprandial MPS was reduced less with whey protein than with soy protein (or carbohydrate (CHO) supplementation) after the intervention, which was predicted to be of importance for the preservation of muscle mass during longer-term energy restriction. Additional support for the use of whey protein during weight loss interventions comes from a study performed in overweight or obese older females on a reduced calorie diet (1400 kcal/d) [78]. During a 6-month intervention, participants received twice-daily whey protein (25 g per serving) supplements or the same does of CHO in the form of maltodextrin. Although no differences were seen in changes to lean mass or muscle strength between the groups, greater weight loss was achieved in the protein group, possibly a consequence of increased satiety and ensuing declines in energy intake [78,79]. In addition, relative to thigh volume changes, the protein group gained ~6% more muscle than the CHO group [78]. In a separate study of older obese individuals on an 8-week weight loss diet, the addition of a 7 g whey protein supplement consumed five times daily did not enhance weight loss, nor did it significantly preserve lean mass [80]. There was however a greater increase in acute postprandial MPS with the protein group [80]. Thus, evidence to date suggests that animal-derived protein feeding during energy restriction can contribute to maintaining muscle health.

To summarise, dietary animal protein does appear to offer benefits to skeletal muscle health in terms of protein turnover, muscle mass and muscle function across the life-course and during both exercise and energy restriction interventions (Table 1).

### 2.2. Bone

Given the importance of protein for bone turnover and matrix remodelling, particularly during growth and ageing [12,13], it is unsurprising that dietary protein has a critical role in modulating bone health. When assessing the effects of animal protein sources on phenotypic (e.g., mass) and functional (e.g., strength) outcomes related to bone health, two recent studies have both reported positive findings. In a cross-sectional study by Durosier et al. [82], bone mineral density (BMD), bone strength and distal radius and tibia bone microstructures were assessed in 746 older females (~65 years). There was a positive association between animal and dairy protein intake with predicted bone failure load (calculated as: force for which 2% of the bone would be loaded beyond 0.7% strain [83]) and stiffness of the distal radius and tibia, which was largely attributed to observed changes in the trabecular bone microstructures. A separate cross-sectional study using dietary intake questionnaire data from >1000 older males from the Osteoporotic Fractures in Men Study, showed positive associations between animal protein intake and bone strength [25]. The findings of each of these studies indicate beneficial effects of animal protein sources on bone strength in older adults.

As previously mentioned, it has also been suggested that diets rich in animal proteins could have negative impacts on bone health [15]. One hypothesis surrounding this relates to the greater acid-forming properties of meat and dairy foods, where it is thought that bone loss could occur through release of salts from the bone to balance the generation of acid [84,85]. Despite this, many studies have found no adverse effects of meat-based protein sources on urinary calcium excretion or other markers of bone health. For example, data from the Framingham Offspring Study found that in 615 older adults, lower protein intake overall was associated with increased bone loss over a 4-year period, while higher intake of animal protein was not associated with decreased BMD [86]. Similarly, in a randomised crossover study of healthy postmenopausal females that directly compared the effects of a high (20% of energy as protein) versus low (12% of energy as protein) meat diet on calcium homeostasis and bone turnover, it was reported that eating a high-meat diet for 16 weeks had no effect on urinary calcium excretion, retention or on circulating markers of bone turnover [87]. A further randomised crossover study also in post-menopausal females studied the effects of a low (10% of energy from protein) versus high (20% of energy from protein) protein and potential renal acid load (PRAL) diet for 7 weeks [88]. The high meat/high PRAL diet led to an increase in both the fractional rate of calcium absorption and urinary calcium excretion, while there was no change in markers of bone resorption or formation. While more evidence is required, these findings indicate that a diet high in animal protein does not adversely affect bone health.

The role of animal-derived protein intake and exercise-induced adaptations on bone health is less studied than protein intake alone. One study by Ballard et al. [89] included young males and females undergoing 6 months of RET and aerobic exercise training, who received a twice daily protein-containing supplementation (84 g/d total protein) or CHO. The protein group had higher plasma insulin-like growth factor-1 levels at the end of exercise training, while serum bone alkaline phosphatase (ALP) also increased with training and tended to be higher in those who received protein. The protein group also had higher concentrations of the bone turnover marker N-terminal telopeptide (NTx). Conversely, during RET in healthy young females, 10 days of high protein (in the form of 2.4 g/kg/d purified whey protein) supplementation during the end of 12 weeks of RET had no effects on bone metabolism, possibly a reflection of the short exercise and supplement period [90]. In relation to advancing age, a study by Holm et al. [91] saw postmenopausal females complete 24 weeks of RET with or without a 10 g whey protein-containing supplement (albeit with calcium and vitamin D) after each training session. The nutrient group had greater increases in BMD as well as increased bone formation (with increased osteocalcin) [91]. Although these effects cannot necessarily be attributed to higher animal protein per se (due to the multi-nutrient supplement), these findings suggest that beneficial effects on bone metabolism can be gained in older adults with long-term training and animal-derived protein provision.

It is generally understood that diet-induced weight loss can have adverse effects on bone health through increased bone resorption [12]. However, the effects of animal protein during weight loss on (markers of) bone health remains poorly studied. One double-blind, randomised, placebo-controlled trial addressed this via whey protein supplementation (20 and ≥40 g) during a combined resistance and aerobic exercise training program in obese/overweight adults [92]. In this study, whey protein, regardless of dose, had no effect on BMD or bone mineral content (BMC) during the intervention. A further trial studied overweight males and females undergoing 12 weeks of energy restriction (6–6.3 MJ/d) with a high-protein (27% of energy from meat, poultry and dairy protein) or standard weight loss diet (16% protein energy) [93]. In this trial, there were no differences in markers of bone turnover or calcium excretion between the groups. A separate study addressed whether a high dairy protein diet containing high calcium (~2400 mg/d) would influence bone turnover during energy restriction in overweight adults [3]. In this study, energy restriction decreased urinary calcium excretion regardless of the calcium content. Following the weight loss intervention, there was an observed increase in bone resorption (determined as an increase in the bone resorption marker deoxypyridinoline), in both groups; however, the diet high in calcium minimised overall bone turnover. Bone health biomarkers were also assessed in a study of pre-menopausal overweight/obese females given differing amounts of dairy protein (dietary protein 30% or 15% of energy) during diet- and exercise-induced weight loss [94]. There was an increase in C-terminal telopeptide of collagen type-I (CTX; a marker for bone turnover), osteocalcin (a marker for bone formation) and urinary deoxypyridinoline in the low (<2% energy from protein) and adequate (dietary protein 15% of energy) protein groups, while no changes in resorption markers but an increase in the bone formation marker amino-terminal pro-peptide of collagen I (P1NP) were seen in the high (30% energy from protein) protein group [94]. These studies indicate that high-protein diets, particularly when higher in calcium, may protect against bone loss during periods of energy restriction and weight loss. Further research is required to directly study the individual effects of protein and calcium on bone health.

In summary, diets high in animal protein appear to be beneficial for bone throughout the lifespan and may offer benefits to bone metabolism in older adults with exercise training. There is also evidence to suggest that animal protein, especially with calcium sufficiency, may counteract some negative effects that weight loss has on bone mass (Table 2).

## 3. Plant-Derived Proteins: Effects in Relation to Age, Exercise, Energy Restriction and Source

### 3.1. Skeletal Muscle

Given the widely reported benefits of animal-derived protein sources on muscle health across the life course, as outlined in Section 2.1 (e.g., References [41,49,50,51]), yet also considering the sustainability of animal- versus plant-derived protein [6], observational studies have assessed the relationship between plant protein consumption and the preservation of muscle health across age. One cohort observational study found that plant-derived protein intake was not positively associated with leg lean mass in older adults, but animal-derived protein was [49]. Additionally, a large (*n* = 2066) longitudinal cohort study of older adults (70–79 years) also showed the importance of protein quality and composition for maintaining muscle mass. In this study, plant protein was not related to a reduced loss of lean mass and appendicular lean mass, however, animal protein was [95]. Similar observations were also seen in a cross-sectional study by Sahni et al. [96] in a wide-ranging age-group (29–86 years), where plant protein intake did not positively associate with leg lean mass, but high total and animal protein intake did. In this study, quadricep strength was greater in the highest plant protein intake quartile comparatively to the lowest quartile, suggesting that sufficient plant protein intake may help to reduce age-related loss of strength.

Despite a lack of convincing evidence from observational cohorts in regard to plant-derived protein (versus animal) and muscle health, a number of studies have gone on to directly compare musculoskeletal-related physiological responses between plant- and animal-derived protein. For example, in healthy young males, ingestion of whey protein stimulated MPS to a greater extent than soy protein, despite matched EAA content [97]. This phenomenon of a diminished MPS response also translates into ageing as Yang et al. [71] demonstrated that, in rested older males, ingestion of either 20 or 40 g whey protein increased MPS, while ingestion of either dose of isolated soy protein elicited no such increases. Furthermore, heightened rates of leucine oxidation were observed in response to ingestion of both 20 and 40 g of isolated soy protein, which may indicate AA oxidation [98]. Similar observations were shown in middle-aged males, whereby postprandial rates of MPS were lower after soy protein ingestion compared to beef [35]. Considering these findings, it may be that consumption of a greater quantity of plant-derived protein may be required to overcome the reduced anabolic response of this protein source. Indeed, a recent investigation by Gorissen et al. [99] in older males demonstrated that although 35 g wheat protein did not stimulate MPS to the same degree as equal amounts of whey or casein protein, when the sources were matched for leucine content (4.4 g), consumption of 60 g wheat protein resulted in a greater MPS response than 35 g whey protein. Interestingly, plasma leucine concentrations increased to a greater extent following whey protein ingestion, with a more gradual appearance of plasma AA after wheat consumption. Sustaining postprandial AA increases may be beneficial in older populations through continued increases in MPS [60], however the practical challenge of getting older adults to consume greater amounts of plant-derived protein to achieve this must be considered given the reported lack of appetite [100] and rapid satiety [101] in older age.

Interestingly, some types of plant-derived proteins (e.g., potato and quinoa) contain adequate amounts of all EAA [9] and thus may offer sufficient anabolic alternatives to animal-derived proteins. Indeed, a recent study in young women found that 25 g of potato protein twice daily for 2 weeks (1.6 g/kg/d total protein) increased integrated MPS above baseline at rest, with no increase observed in those consuming a control diet (0.8 g/kg/d total protein) [102]. Whilst this greater anabolic response could be attributed simply to the greater amount of protein, it still demonstrates the ability of potato protein to stimulate MPS above a baseline diet already containing the RDA of protein, at least in younger individuals. Further, since it is well recognised that plant-derived protein sources can have inferior anabolic properties compared to animal-derived protein sources, the notion of blending different plant-derived sources together (in order to exploit the favourable AA profile of each) has been suggested in order to improve the anabolic quality of plant-derived protein sources. Protein blends, including plant–plant protein blends, are discussed in more depth in Section 5.

Considering protein × exercise interactions, it has been shown that combining whey protein ingestion with RE/T capitalises upon postprandial stimulation of MPS responses, therein promoting gains in muscle mass and strength [71,97]. However, the efficacy of plant-derived (as opposed to animal-derived) protein to potentiate exercise-induced anabolism is less well studied. In the context of acute exercise, whey protein ingestion in conjunction with unilateral RE elicited a greater MPS response than that of an EAA-matched (10 g) soy protein in young males [97]. However, both whey and soy protein increased MPS rates to a greater extent than casein protein in both rest and exercise conditions. This may be due to the slower nature of casein digestion and subsequent aminoacidemia, with whey protein increasing aminoacidemia to a more rapid and greater degree than intermediary soy protein [97]. Conversely, comparable MPS stimulation was observed post-exercise in those consuming potato protein (25 g twice daily) and control diet groups over a 2-week period, highlighting the potency of RE as an anabolic stimulus [102]. In the context of ageing, a randomised cross-over study by Wilkinson et al. [103] found that the ingestion of soy protein (18.2 g) with acute RE increased MPS responses to a lesser degree than that of isonitrogenous whey protein in young males. This was despite greater total plasma AA and similar leucine concentrations following consumption of soy protein.

In regard to chronic exercise × plant-protein interactions, a 6-week whole-body RET (3 d/week) intervention involving supplementation of whey or soy protein (1.2 g/kg, consumed as three equal doses per day) increased lean mass and strength in young adults to a greater degree than an isocaloric maltodextrin placebo [104]. Furthermore, no differences between the protein groups were observed, and fractional breakdown rate remained constant throughout, suggesting greater MPS, independent of protein source. Conversely, some have reported no effects of whey or soy protein on muscular adaptations to RET [105] and others have shown that compared to milk, soy protein induced inferior gains in muscle hypertrophy in young males [106]. Interestingly, other plant-derived protein sources have demonstrated similar benefits when combined with RET. For example, twice daily ingestion of pea or whey protein (26.6 g protein, 2.9 g leucine and 23.9 g protein, 3.9 g leucine, respectively) combined with progressive upper-body RET each improved bicep thickness after 42 and 84 days in young males [107]. Furthermore, sub-analysis of weaker (at study start) adults showed that consuming pea protein increased muscle thickness to a greater degree than whey protein or placebo. Similarly, consumption of rice protein isolate (48 g protein, 3.84 g leucine, 3 ×/week) during 8 weeks of whole-body RET improved lean mass gains and body composition to a comparable extent as isonitrogenous whey protein (48 g protein, 5.5 g leucine) in young males [23]. In a study of older males, the addition of a diet high in beef or soy protein (0.6 g protein/kg/d from beef or soy, respectively) to whole-body RET for 12 weeks, each increased strength and *m. vastus lateralis* cross-sectional area to a similar extent [81]. Interestingly, the comparable increases in skeletal muscle mass independent of protein source in a number of these studies [23,81] may be a result of consuming greater protein amounts (and subsequently leucine), thereby offsetting the often reduced EAA content with plant-derived proteins and supporting augmentation of RET-induced gains in lean mass. Thus, sustained consumption of greater quantities of plant-derived proteins in conjunction with RET may be sufficient to support increases in muscle mass (Table 3).

### 3.2. Bone

Despite plant-derived proteins varying in AA composition depending on the plant protein source (i.e., corn and wheat), similar sulphur content has been reported across these sources [110] suggesting possibly ubiquitous effects on bone health. Although limited studies directly comparing the effects of protein sources on bone health are available, a recent meta-analysis by the National Osteoporosis Foundation was undertaken evaluating the influence of differing protein source supplementation on the bone health of healthy adults [111]. This analysis of randomised controlled trials concluded that supplementation of either soy or animal protein for >1 year was beneficial on multiple outcomes of bone health (BMD in lumbar spine, total hip, femoral neck and total body), with neither more advantageous than the other. Moreover, a separate randomised controlled trial by Dawson-Hughes et al. [2] showed that during a 3-year supplementation period of calcium and vitamin D in older males and females, greater protein intake (irrespective of the source) was associated with increased BMD. It is important to note that such improved bone-related outcomes resulting from increased protein intake require sufficient dietary calcium intake, and the relationship between protein intake and BMD was not observed in the control group for this study [2]. Despite these findings, translation of the results from these supplementation studies into additional clinically relevant outcomes remains unclear. For instance, one recent cohort study showed that greater protein intake of animal protein was associated with reduced risk of hip fracture in older males, whereas plant-derived protein was not [24], which could be related to the higher calcium content in animal-derived versus plant-derived protein sources. Comparatively, in a separate 5-year cohort study of older males and females, greater protein intake was associated with reduced fracture risk, but this was not related to protein source [112].

Variable findings on the effect of protein sources on bone health may result from additional constitutive elements present, such as isoflavones, which are predominantly present in soy protein products. In support of this, epidemiological studies have associated a decreased risk of bone loss and hip fracture risk in older Asian populations with consuming proportionally more soy protein [113]. Structurally similar to oestrogens, isoflavones have been demonstrated to reduce bone turnover through a combination of stimulating bone formation and inhibiting bone reabsorption [114,115]. Furthermore, isoflavone inclusion rather than protein alone may be an important aspect for bone health, as in a 24-week supplementation period in perimenopausal women, only supplementation with isoflavone-rich soy was able to attenuate losses in BMD and BMC when compared to isoflavone-poor soy protein or whey protein control [116]. However, results from isoflavone supplementation studies have been inconsistent, with supplementation of isoflavone-enriched products (110 ng/d) alongside habitual diets for 1 year not shown to prevent postmenopausal bone loss [117], suggesting that increased protein consumption may also be needed. That said, soy isoflavanols’ (70 mg/day) supplementation increased bone formation markers (i.e., bone-specific ALP and osteocalcin), whilst reabsorption markers remain unchanged (i.e., CTX and NTx) [118]. Although further investigation is required to elucidate potential benefits of isoflavones and corresponding protein supplementation, high habitual soy protein intake (containing isoflavones) may be beneficial for the maintenance of bone health and/or the attenuation of bone loss.

To summarise, plant-derived dietary protein has the potential to induce similar anabolic responses to animal-derived protein, particularly when matched for leucine, in the context of acute and chronic exercise. Additionally, plant protein alone (i.e., not in the context of exercise/energy restriction) demonstrates beneficial effects on certain aspects of bone health (e.g., BMD), although this may be in part due to the effects of plant protein containing isoflavanols (Table 4).

## 4. Collagen-Derived Proteins: Effects in Relation to Age, Exercise, Energy Restriction and Source

### 4.1. Skeletal Muscle

Collagen proteins are the most abundant proteins in the human body [121], accounting for ~25–30% of total protein body mass [26], and are the major constituents of many tissues, including connective tissue, tendons, ligaments and bones [122]. Thus, dietary collagen is likely a key mediator of musculoskeletal remodelling throughout the lifespan. As such, collagen supplementation, in the form of collagen hydrolysates or gelatin, has recently gained popularity as an alternative or adjunct protein source to animal- and/or plant-derived sources for maintaining or even potentiating muscle and/or bone health (i.e., mass/function). This may seem counterintuitive since dietary collagen is rich in non-essential amino acids (NEAA’s; e.g., proline, glycine), low in EAA’s (e.g., methionine, leucine) and lacks tryptophan, rendering a DIAAS of 0 [27]. Expectedly, this has led to some questioning the anabolic potential of dietary collagen, at least compared to high-quality protein sources such as whey protein, which contain high levels of leucine and have a DIAAS of >1 [123]. Nevertheless, pre-clinical models have shown collagen-specific peptides to offset disease-induced muscle wasting [124], inhibit age-related muscle oxidative decline [125] and promote muscle hypertrophy via increased mTOR signalling [122], therein demonstrating the anabolic potential of supplemental collagen-derived proteins. This, coupled with the fact that dietary collagen has superb digestibility and becomes rapidly bioavailable following consumption in humans [28,126], suggests that there is potential for dietary collagen to mediate human skeletal muscle and bone remodelling. However, to date, the effects of collagen supplementation on muscle health across age, in the absence of allied exercise, has been sparsely studied.

In regard to ageing, older females consuming the RDA of protein with collagen constituting approximately half of the total protein provided, preserved lean body mass and maintained nitrogen balance [127]. In contrast, those consuming a similar quantity of whey protein experienced a loss in body weight with no change in body fat (potentially indicating a decline in lean body mass) and an increase in nitrogen excretion [127]. Despite collagen being regarded as a low-quality protein (according to PDCAAS and DIAAS scores), the NEAA’s it does contain either have a low molecular weight or possess more than one nitrogen atom (e.g., hydroxyproline, hydroxylysine), meaning the nitrogen content of collagen on a per gram basis is high [8], and possibly greater than whey protein [127], which may explain the ability of collagen to help maintain nitrogen balance.

The ability of collagen supplementation to potentiate exercise-induced muscle adaptations is more widely studied than the effects of collagen supplementation alone yet remains contentious with mixed results depending on the outcome measure. In regards to body composition, Kirmse et al. [128] observed an increase in fat-free mass after 12 weeks of RET plus 15 g/d collagen peptide supplementation, which was not observed in the placebo group. However, similar changes in cross-sectional area and muscle thickness across the whole cohort (i.e., both groups) suggest that greater myofiber hypertrophy cannot explain these changes. Other data shows blunted RE-induced increases in anabolic signalling (p70S6K) (collagen vs. whey protein) [129] and muscle sub-fraction (myofibrillar and sarcoplasmic) MPS (collagen vs. α-lactalbumin) [130], with collagen protein, albeit in the context of short-term (3-days) aerobic exercise. The lack of tryptophan and low methionine and leucine content [27] in dietary collagen may explain the non-hypertrophic responses when used in an unblended fashion (i.e., when not blended with other dietary protein sources). Instead, it has been suggested that increased connective tissue/extracellular matrix (ECM) remodelling may contribute to the favourable changes in fat-free mass that are observed [128]. This supposition is supported by data showing that gelatin supplementation increased collagen content in engineered ligaments [126]. Similar mechanisms may also underlie muscle functional responses, specifically muscle strength, where studies have shown collagen peptide supplements to have no effect on maximal voluntary contraction [29,128], but did speed-up recovery of countermovement jump performance following strenuous exercise [29]. Since ECM degradation can occur following exercise [131], it is plausible that the purported collagen-induced ECM remodelling (e.g., increased collagen synthesis) occurred, therein improving fast/reactive movements that have a heavy tendon component (i.e., countermovement jump) [128]. Further, the ability of collagen supplementation to facilitate the recovery of additional exercise performance measures, such as maximal voluntary contraction, following an intense period of short-term RET was similar to that of whey protein [132].

In the context of exercise and ageing, Zdzieblik et al. found that RET for 12 weeks combined with 15 g/d collagen peptide supplementation led to substantial increases in fat-free mass (+4.2 kg) and decreases in fat mass (−5.5 kg) in sarcopenic males [26]. However, similar intervention studies using potent and established nutritional (e.g., animal-derived protein [5]) and pharmacological (e.g., testosterone [133]) stimulators of muscle growth did not observe such marked increases in fat-free mass [134], leading some to question the findings of Zdzieblik et al. [134]. More recently, Jendricke et al. [135] also reported greater increases in fat-free mass and a greater decrease in fat mass following dietary collagen supplementation, supporting positive body composition changes in response to this form of supplement. In the absence of known mechanisms, it has been suggested that a reduction in adipocyte size may contribute to changes in fat mass [121], in addition to the already mentioned hypothesis of ECM adaptations contributing to fat-free mass gains. That said, collagen peptide supplementation in older females did not increase rates of integrated collagen (or myofibrillar) MPS above baseline or in response to two bouts of RE [27], contradicting the suggestion of impacts on ECM remodelling (at least in older females). Zdzieblik et al. [26] also proposed that collagen-induced creatine synthesis may underlie changes in fat-free mass, yet daily provision of arginine and glycine from collagen is small, and thus this has been refuted as a potential mechanism [134]. Collagen supplementation has also been tested in the context of blood flow restriction, an exercise modality shown to induce favourable changes in muscle mass in the context of low-intensity RET (~20–30% of 1 repetition maximum) [136,137]. In older males, collagen hydrolysate supplementation for 8 weeks adjunct to low-load blood flow restriction RET tended to increase muscle cross-sectional area (+6.7%) more than placebo (+5.7%). Although this was only reported as a trend (i.e., non-significant), this is likely due to the low participant numbers (*n* = 11 in each collagen and placebo group), and thus further studies are required to substantiate these findings.

In the only study of its kind (to date) involving collagen supplementation, energy restriction (500 kcal/d reduction) and subsequent energy restriction plus activity reduction led to incremental declines in myofibrillar MPS, which increased during return to habitual activity when supplemented (throughout) with 30 g whey protein but not with isonitrogenous and isoenergetic collagen protein [77]. In the same study, both energy and energy plus physical activity restrictions (≤750 steps/d) led to reduced lean body mass and leg lean mass, neither of which were mitigated with either collagen or whey protein supplementation, despite protein consumption amounting to twice the RDA for protein [77].

To summarise, dietary collagen does not appear to stimulate MPS in the context of ageing and/or exercise. However, there is evidence to suggest that it can promote favourable body composition and muscle functional adaptations when combined with exercise, independent of age, possibly mediated by ECM remodelling. It is therefore plausible that collagen protein provided simultaneously with nutritional stimulators of myofiber hypertrophy (i.e., animal/plant protein sources), may maintain and/or potentiate muscle health via dual mechanisms targeting both ECM and myofiber remodelling (Table 5). 

### 4.2. Bone

The anabolic effects of collagen supplementation on bone health have been recognised in pre-clinical models demonstrating enhanced bone metabolism [138], microarchitecture [139] and offsetting age-related bone density decline [125], although the effects in humans are less well understood. In the context of ageing, post-menopausal females supplemented with 5 g/d calcium-collagen chelate (albeit with 500 mg calcium and 200 IU vitamin D_3_) for 12 months had attenuated whole-body BMD losses compared to control (calcium and vitamin D_3_) [140]. Whilst these positive effects cannot necessarily be attributed purely to collagen supplementation due to insufficient dietary control, these findings were later echoed by a 12-month randomised, double-blind, placebo-controlled trial, in which postmenopausal females given 5 g/d collagen peptide demonstrated significant increases in BMD of the femoral neck and lumbar spine and an increase in P1NP, indicative of an increase in bone formation [141]. Expectedly, the control group (5 g/d maltodextrin) displayed numerical (non-significant) declines in BMD and increases in CTX, indicative of bone degradation over the intervention period [141]. Since there were no differences in macro- or micro-nutrient intake observed between the treatment and control groups pre- or post-intervention, this may indicate that the positive effects on BMD are attributable to collagen supplementation.

In the context of exercise, it is thought that the liberation of collagen-specific AA (e.g., glycine, proline) from dietary collagen may potentiate exercise-induced collagen synthesis, therein facilitating bone remodelling. This hypothesis is somewhat supported by data from Shaw et al. [126], who found that supplementation with 5 g of vitamin-C (48 mg)-enriched gelatin (denatured form of collagen [122]) prior to intermittent high-impact exercise in young males increased circulating P1NP, indicative of increased bone collagen synthesis. Outwardly, this makes sense as vitamin-C is a cofactor for the enzymes lysyl hydroxylase and prolyl hydroxylase, which are essential for collagen synthesis [142]. In the absence of vitamin-C, 20 g/d collagen supplementation before and after strenuous exercise in young males had no significant effects on P1NP or β-isomerised CTX [29], suggesting that collagen alone does not support bone collagen synthesis. The discrepant findings between these studies may be due to the vitamin-C content or based on other methodological differences such as the differing collagen source (i.e., collagen peptide vs. gelatin) and/or amount (i.e., 20 g/d vs. 15 g/d vs. 5 g/d) of collagen provided.

The effects of collagen supplementation in tandem with chronic exercise training on bone health are not yet well established. In older sarcopenic males, 6 weeks of RET with 15 g/d collagen peptide supplementation did not augment RET-induced gains in bone mass [26], although the authors do not speculate about the reason for this lack of effect. Since one year of collagen supplementation alone (i.e., in the absence of RET) did improve BMD in older adults [141], it is conceivable that the supplementation period was too short to elicit collagen-induced effects in the slow turnover tissue that is bone.

Overall, dietary collagen appears to offer benefits to bone health in terms of offsetting age-related bone loss, potentially mediated by increasing bone formation and decreasing bone degradation. However, the synergistic effects of collagen supplementation and exercise remain contentious (Table 6).

## 5. Animal-, Plant- and/or Collagen-Derived Protein Blends

Protein blends offer a potentially viable and sustainable option to compensate for EAA deficiencies in some protein sources, thereby overcoming the inferior anabolic profiles of plant- and collagen-, versus animal-derived protein. Most research to date has assessed the effects of animal and plant blends in the context of skeletal muscle health, where the combination may exploit the digestive properties of each protein source, maximising AA availability and potentially extending and augmenting the MPS response [9]. For example, a protein blend containing 25% whey protein, 25% soy protein and 50% casein protein provided after acute RE in young healthy males stimulated mixed MPS in a similar fashion to whey protein [30]. Considering both supplements provided similar amounts of EAA and leucine, the similar anabolic stimulus is unsurprising and thus provides evidence to support the use of plant-derived proteins to promote acute muscle anabolism alongside animal-derived sources. This similar anabolic response to animal and plant blends (versus whey protein) also holds true in older adults [32]. However, it should be noted that in both of these studies, only 25% of the protein blend was plant-derived, and since plant-derived protein contains lower leucine and less EAA, the consumption of protein blends with higher percentages of plant-derived sources may not be as effective for stimulating muscle anabolism, particularly in older adults who display anabolic resistance [7,55], although this remains to be determined. Current evidence in regard to protein blends × exercise interactions is sparse, however, daily supplementation of a soy and dairy protein blend (containing 25% whey protein, 25% soy protein and 50% casein protein) during whole-body RET for 12 weeks tended to increase lean body mass compared to maltodextrin control, with no trend observed in a whey protein supplemented group [31]. Whilst this may be a reflection of the beneficial divergent digestive properties of these protein sources, further research is required to confirm or refute this.

Although the consumption of animal and plant protein-blends may be suitable for some, they will not be suitable for all (i.e., vegans) and as such, sustainable plant- and plant-derived protein blends (i.e., blending two or more different plant protein sources) are an emerging area of interest and research. Whilst there is no experimental evidence available to date, it is plausible that combining a plant-derived protein source low in lysine and high in methionine (e.g., rice) with another plant-derived protein source with a divergent EAA profile (e.g., pea protein) will provide a plant protein blend that satisfies all of the EAA necessary for robustly stimulating MPS [7,9]. Indeed, researchers have started to develop a variety of mixed plant-protein blends that exceed current AA requirements [9]. However, whether these blends stimulate MPS similarly to animal-derived proteins remains to be seen [9]. Further, the efficacy of plant and collagen protein blends for muscle health (both with and without animal-derived protein) warrants future research, particularly since collagen may support ECM remodelling and thus may be particularly effective in the context of acute/chronic exercise.

In summary, the limited available evidence suggests that animal and plant protein blends may support anabolic responses to acute exercise.

## 6. Future Directions

Despite a wealth of protein source research to date, many gaps remain in our understanding of how animal, plant, collagen and blended protein sources can modulate musculoskeletal outcomes, particularly in the context of ageing, exercise and energy restriction. Here, we outline some of the key gaps that we suggest are worthy of imminent future investigation.

Whilst animal-derived protein sources are by far the most investigated protein source to date, the length of time that the muscle remains refractory to dietary animal-derived protein following the onset of “muscle full” remains to be determined. Whether the kinetics of this refractory period (e.g., duration) are dependent on the protein source (i.e., plant, collagen, blended) also remains to be investigated thoroughly. Further, whilst much research has investigated the optimal (e.g., amount, type, etc.) animal-derived protein feeding strategy, the optimal type, texture, matrix and amount of animal protein that is most beneficial for maintaining and potentiating musculoskeletal health (i.e., muscle and bone) during energy restriction remains to be fully investigated.

Similarly, there is a lack of rigorous experimental findings in regard to the effects of plant-derived protein sources on both muscle and bone health during energy restriction, warranting further investigation.

In regard to collagen protein, further research should look to substantiate previous evidence suggesting that collagen protein may support blood flow restriction RET-induced muscle growth. In the context of bone health, collagen protein provided in conjunction with vitamin-C may herald anabolic bone remodelling effects, although this remains to be investigated. Since much less research exists regarding collagen protein (compared with animal- and plant-derived protein), much more work is needed to determine the optimal collagen protein dosing strategy (e.g., amount, timing), including adjuvant nutritional needs (i.e., vitamin-C, protein blends), that is most beneficial for potentiating muscle and bone health, particularly in the context of exercise and energy restriction.

Finally, whilst there is evidence to suggest that protein blends may support musculoskeletal remodelling in response to acute exercise, further evidence is required to clarify the effects in regard to supporting chronic RET-induced musculoskeletal adaptations. Theoretically, mixed plant, plant and collagen, animal and collagen, and animal and plant blends each have anabolic potential, however, the most sustainable and efficacious protein blend for anabolic stimulation in the context of exercise, ageing and energy restriction remains to be determined.

## 7. Conclusions

**Figure 1:**
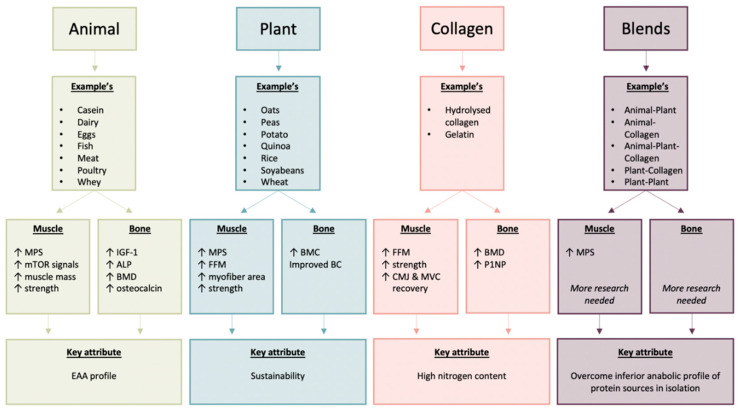
Summary of the key musculoskeletal benefits of animal, plant, collagen and blended dietary proteins. Abbreviations: ALP, alkaline phosphatase; BC, body composition; BMC, bone mineral content; BMD, bone mineral density; CMJ, counter movement jump; EAA, essential amino acids; FFM, fat free mass; IGF-1, insulin-like growth factor 1; MPS, muscle protein synthesis; mTOR, mechanistic target of rapamycin; MVC, maximal voluntary contraction; P1NP, amino-terminal propeptide of collagen I.

In conclusion, an increased appreciation of the role of protein sources (including protein blends) in relation to the musculoskeletal system under beneficial (e.g., exercise) and deleterious (e.g., ageing, energy restriction) perturbations is crucial to informing appropriate nutritional support in the face of complex challenges, such as changes in appetite and/or socio-economic trends. Plant-derived proteins may provide suitable alternatives to animal proteins in relation to musculoskeletal health, albeit under some circumstances at higher-intakes. Similarly, collagen-derived proteins represent relatively nitrogen-dense sources that have shown some efficacy in relation to favourable body composition changes. Protein blends harnessing the biological benefits of distinct protein sources may represent a means by which to maximise the health benefits of dietary proteins in relation to musculoskeletal health. A schematic representation of this conclusion is presented in Figure 1.

## Figures and Tables

**Table 1:** Animal-derived proteins: effects on muscle in relation to age, exercise, energy restriction and source.

Reference	Study Design	Protein Composition	Measurements	Key Outcomes
Alexandrov et al., 2018 [49]	Data analysis of the Lifelines Cohort 31,278 males (M) and 45,355 females (F) (*n* = 76,633, 44.9 ± 12.8 years, 18–91 years) (mean ± standard deviation (SD))	Protein type/intake determined through food frequency questionnaire (mean protein intake per day 1 ± 0.3 g/kg)	Protein intake, muscle mass (24 h urinary creatinine excretion)	Increased intake of total and animal protein associated with increased creatinine excretion in M and F
Bradlee et al., 2018 [1]	Data analysis of the Framingham Offspring study Diet, physical activity and functional performance data collected from M (*n* = 1016) and F (*n* = 1333) to evaluate effects on muscle mass	Protein type/intake determined through 3-day food records	Dietary analysis, physical activity, % muscle mass, functional performance	Higher protein intake associated with higher % muscle mass over a 9-year period Higher intake of animal protein had higher % muscle mass In those less active, only animal protein consumption reduced risk of functional decline
Symons et al., 2009 [41]	Healthy young adults (M *n* = 8, F *n* = 9, 35 ± 3 years) and older (M *n* = 10, F *n* = 7, 68 ± 2 years) randomly assigned to moderate or large protein serving (mean ± SD)	Single moderate serving (113 g; 220 kcal; 30 g protein) of 90% lean beef Large (340 g; 660 kcal; 90 g protein) serving of 90% lean beef	Muscle protein synthesis (MPS)	Moderate serving of beef increased MPS ~50% in young and older adults with no further increase seen after ingestion of a large serving
Alemán-Mateo et al., 2014 [66]	Single-blind randomised controlled trial (RCT) Older adults randomised to habitual diet (M *n* = 25, F *n* = 25, 69.6 ± 6.4 years) or habitual diet with ricotta cheese (M *n* = 25, F *n* = 25, 70.8 ± 7.6 years) for 12 weeks (mean ± SD)	12 weeks of habitual diet or habitual diet with dairy-rich protein (210 g ricotta cheese)	Lean mass (LM), muscle strength	LM increased in supplemented group relative to normal diet group Both groups lost strength but greater loss of muscle strength in controls
Zhu et al., 2015 [67]	Randomised, double-blind, placebo-controlled design F were randomly assigned to a high protein drink (*n* = 101, 74.2 ± 2.8 years) or placebo (*n* = 95, 74.3 ± 2.6 years) (mean ± SD)	Over a 2-year period, F consumed either daily high protein drink (30 g of whey protein) or placebo (2.1 g protein)	Appendicular lean mass (ALM), muscle cross-sectional area (CSA), handgrip strength, lower limb muscle strength, dietary analysis	Both groups showed decrease in upper arm and calf muscle area over 2 years, but no change in ALM No effect of protein supplementation on muscle mass or function after 1 or 2 years
Luiking et al., 2014 [73]	RCT Healthy older adults were randomised to consume either high whey protein (*n* = 9, 66.9 ± 4.8 years) or milk protein control (*n* = 10, 71.1 ± 6.3 years) after unilateral resistance exercise (RE) (mean ± SD)	Single bolus of high whey protein, leucine-enriched supplement containing 20 g whey protein, 3 g total leucine Isocaloric milk protein control containing 6 g milk protein	MPS, dietary analysis	Higher MPS with whey protein supplement than milk protein
Witard et al., 2014 [44]	Single-blind parallel design Young, resistance-trained M (*n* = 48) were randomised to consume 0 (22 ± 3 years), 10 (20 ± 1 years), 20 (22 ± 3 years) or 40 (20 ± 1 years) g protein after a single bout of unilateral RE (mean ± SD)	0, 10, 20 or 40 g whey protein isolate following RE	MPS, whole-body phenylalanine oxidation, dietary analysis	Ingestion of 20 and 40 g whey protein increased myofibrillar MPS above 0 g 40 g whey protein increased rates of phenylalanine oxidation
Farnfield et al., 2012 [74]	Randomised, placebo-controlled design Healthy young and older M completed a 12-week resistance exercise training (RET) and were randomly assigned to consume whey protein (young *n* = 8, 20.5 ± 0.7 years, older *n* = 9, 68.1 ± 1.6 years) or placebo (young *n* = 8, 20.4 ± 0.8 years, older *n* = 9, 67.4 ± 1.3 years) after each exercise session (mean ± standard error of the mean (SEM))	Whey protein containing 26.6 g amino acids (AA) per serving Placebo containing same amount of artificial flavour and aspartame sweetener	Strength, protein signalling, dietary analysis	Strength increased in all volunteers Whey protein caused greater increases in mechanistic target of rapamycin phosphorylation than placebo in both age groups
Robinson et al., 2013 [72]	RCT 35 M (59 ± 2 years) were randomly assigned to 1 of 4 protein groups with and without RE (*n* = 7 per group) (mean ± SEM)	Consumed 0, 57 g (12 g protein), 113 g (24 g protein) or 170 g (36 g protein) of ground beef	MPS, leucine oxidation	Ingestion of 170 g beef increased myofibrillar MPS at rest and after RE more than other amounts Higher leucine oxidation with increasing amounts of beef
Yang et al., 2012 [71]	RCT Older M (*n* = 37, 71 ± 4 years) completed a bout of unilateral leg RE prior to ingesting 1 of 4 protein doses (mean ± SD)	0, 10, 20 or 40 g whey protein isolate	MPS, leucine oxidation	Whole-body leucine oxidation increased in a dose-dependent manner MPS increased with 20 and 40 g whey protein but not lower doses 20 and 40 g whey protein ingestion post-exercise increased MPS above 0 and 10 g exercise rates
Haub et al., 2002 [81]	RCT M randomly assigned to beef-containing (*n* = 10, 63 ± 3 years) or lacto-ovo-vegetarian (*n* = 11, 67 ± 6 years) diet throughout 12-week RET (mean ± SD)	Beef-containing diet: 0.6 g protein/kg/d from beef Lacto-ovo-vegetarian diet: 0.6 g protein/kg/d from soy	Strength, muscle CSA, dietary analysis	No difference between dietary groups in terms of strength improvements CSA of *m. vastus lateralis* increased with training similarly in both groups
Chalé et al., 2013 [75]	Randomised, double-blind controlled design Older mobility-limited adults were randomised to protein (*n* = 42, 78 ± 4 years) or isocaloric control (*n* = 38, 77.3 ± 3.9 years) and high intensity RET for 6 months (mean ± SD)	Whey protein: 40 g/d Isocaloric control	Strength, muscle CSA, LM, dietary analysis	LM, muscle CSA and muscle strength increased in both groups but there was no difference between groups
Kang et al., 2019 [76]	Multicentre, interventional, two parallel-group case-control design Frail older adults received daily protein supplementation (*n* = 49, 78 ± 7 years) or no supplementation (*n* = 66, 76.8 ± 7 years) combined with RET for 12 weeks (mean ± SD)	Protein containing 32.4 g of whey protein	Handgrip strength, gait speed, chair rise test	Handgrip strength, chair-stand time and gait speed improved to a greater extent in the group that received whey protein
Hector et al., 2015 [4]	Randomised, double-blind design Adults were randomised to receive whey protein (*n* = 14, 52 ± 2 years), soy protein (*n* = 14, 52 ± 2 years) or carbohydrate (CHO) (*n* = 12, 48 ± 3 years) during a 14-day hypoenergetic diet (mean ± SEM)	Twice daily supplements of: Whey protein: 27 g/supplement or Soy protein: 26 g/supplement or Isoenergetic CHO Hypoenergetic diet: −750 kcal/d	MPS, dietary analysis	Whey protein stimulated MPS to greater extent than soy protein or CHO pre-intervention Postprandial MPS was reduced by whey protein less than soy protein and CHO post-intervention
Mojtahedi et al., 2011 [78]	Randomised, double-blind parallel design Overweight/obese, postmenopausal F prescribed reduced calorie diet and randomised to protein (*n* = 13, 64.7 ± 4.4 years) or CHO (*n* = 13, 64.6 ± 5.2 years) for 6 months (mean ± SD)	Reduced calorie diet: 1400 kcal/d, 15%, 65% and 30% energy from protein, CHO and fat, respectively. Protein: 2 × 25 g/d whey protein CHO: 2 × 25 g/d maltodextrin	LM, strength	More weight lost in protein group No differences changes to LM or strength Relative to thigh volume changes, protein group gained more muscle than CHO group
Coker et al., 2012 [80]	Older adults (*n* = 12) randomised to 8-week calorie restriction diet using (i) 7% weight loss with meal replacement (70 ± 2 years) or (ii) competitive meal replacement (68 ± 2 years) (mean ± SEM)	Whey protein (7 g) plus essential amino acids (EAA) formulation (6 g) in form of meal replacement (5 ×/d) or competitive meal replacement	LM, MPS, dietary analysis	Whey protein/EAA did not preserve LM but there was an increase in acute FSR

Abbreviations: AA, amino acids; ALM, appendicular lean mass; CHO, carbohydrate; CSA, cross-sectional area; EAA, essential amino acids; F, females; LM, lean mass; M, males; MPS, muscle protein synthesis; RCT, randomised controlled trial; RE, resistance exercise; RET, resistance exercise training; SD, standard deviation; SEM, standard error of the mean; d, day.

**Table 2:** Animal-derived proteins: effects on bone in relation to age, exercise, energy restriction and source.

Reference	Study Design	Protein Composition	Measurements	Key Outcomes
Hannan et al., 2000 [86]	615 older adults (75 ± 4.4 years, 391 females (F), 224 males (M) (mean ± standard deviation (SD)) Relationship between dietary protein and subsequent 4-year change in bone health	Protein type/intake determined through food frequency questionnaire	Protein intake, bone mineral density (BMD)	Lower protein intake associated with increased bone loss Higher intake of animal protein not associated with decrease in BMD
Roughead et al., 2003 [87]	Randomised crossover design Healthy postmenopausal F (*n* = 15, 60.5 ± 7.8 years) randomised to 8-week high-meat and 8-week low-meat diet (mean ± SD)	High-meat diet: 20% of energy as protein Low-meat diet: 12% of energy as protein Calcium content similar (~600 mg) in both diets	Calcium excretion, bone markers, dietary analysis	High-meat diet did not adversely affect urinary calcium excretion, calcium retention or markers of bone metabolism
Cao et al., 2011 [88]	Randomised crossover design Postmenopausal F (*n* = 16, 56.9 ± 3.2 years, mean ± SD) randomised to two diets: low protein, low potential renal acid load (PRAL) and high protein, high PRAL diet.	Low protein, low PRAL diet: 10% of energy as protein High protein, high PRAL diet: 20% of energy as protein Each diet was 7 weeks separated by 1 week	Calcium absorption, bone markers, dietary analysis	No effect of high meat/PRAL diet on markers of bone metabolism Increased fractional rate of calcium absorption and urinary calcium excretion
Durosier-Izart et al., 2017 [82]	Cross-sectional study design 746 F (65 ± 1.4 years, mean ± SD) Associations between animal (separated into non-dairy and dairy) and vegetable protein sources and bone health	Protein type/intake determined through food frequency questionnaire	Areal BMD, distal radius and tibia bone microstructures, bone strength, protein intake	Predicted failure load and stiffness at distal radius and tibia positively associated with total, animal and dairy protein intake
Langsetmo et al., 2018 [25]	Cross-sectional study design Questionnaire data from 1016 M (84.3 ± 4 years, mean ± SD) Association of dairy, non-dairy and plant-derived protein intake on bone health	Protein type/intake determined through food frequency questionnaire	Bone strength, BMD, protein intake	Higher dairy protein associated with higher estimated failure load at the distal radius and distal tibia Higher non-dairy animal protein associated with higher total BMD
Ballard et al., 2006 [89]	Randomised controlled trial 51 younger adults (18–25 years, 28 M, 23 F) were randomised to either protein (20.9 ± 2.4 years) or placebo (21.1 ± 2.2 years) supplementation during a 6-month training intervention of alternating resistance exercise training (RET) and aerobic exercise 5 ×/week (mean ± standard error of the mean (SEM))	Twice daily protein (42 g protein, 24 g carbohydrate (CHO), 2 g fat) Isocaloric CHO supplement (70 g CHO)	Bone markers, protein intake	Increases in plasma insulin-like growth factor-I greater in protein group Serum bone alkaline phosphatase increased over time and tended to be higher in protein group N-terminal telopeptide concentrations greater in protein group
Mullins & Sinning, 2005 [90]	Randomised, double-blind, placebo-controlled design 24 healthy, untrained, young adult F (18–29 years) engaged in 12-week RET 3 d/week and were randomised to protein (22.8 ± 0.9 years) or placebo (22.7 ± 1.1 years) during the final 10 days (mean ± SEM)	High-protein diet (during final 10 days): purified whey protein for daily protein intake of 2.4 g/kg/d Control: equivalent dose of isoenergetic CHO	Bone markers, dietary analysis	High protein intake for final 10 days of RET had no effects on bone metabolism
Holm et al., 2008 [91]	Randomised, double-blind, placebo-controlled design Postmenopausal F were randomised to a protein-containing nutrient supplement (*n* = 13, 55 ± 1 years) or placebo (*n* = 16, 55 ± 1 years) in conjunction with 24-week RET (mean ± SEM)	Nutrient supplement containing: 10 g whey protein, 31 g CHO, 1 g fat, 250 mg calcium and 5 µg vitamin D. 730 kJ in total. Placebo supplement containing: 6 g CHO and 12 mg calcium. 102 kJ in total. Supplements were consumed after each training session	BMD, bone markers, dietary analysis	Nutrient group had greater increase in BMD at the femoral neck than controls Increased bone formation and osteocalcin following training in nutrient group
Wright et al., 2017 [92]	Randomised, double-blind, placebo-controlled design Obese/overweight adults were randomised to 0 g protein (*n* = 68, 50 ± 7 years) 20 g protein (*n* = 72, 48 ± 8 years) or ≥40 g protein (*n* = 46, 49 ± 8 years) combined with 36-week RET and aerobic exercise training 3 d/week for 36 weeks (mean ± SD)	Unrestricted diet in combination with whey protein supplementation (0, 20, 40 or 60 g/d) (40 and 60 g group combined to form a ≥40 g group for analysis)	BMD, bone mineral content (BMC), protein intake	Whey protein, regardless of dose, had no effect on BMD or BMC during training
Farnsworth et al., 2003 [93]	Parallel design 57 overweight adults randomised to either high protein (M *n* = 7 51.9 ± 3.3 years, F *n* = 21, 50.6 ± 2.7 years) or standard protein (M *n* = 7 48.6 ± 3.2 years, F *n* = 22, 50.6 ± 2.1 years) diet during 12 weeks of energy restriction and 4 weeks of energy balance (mean ± SEM)	High-protein diet of meat, poultry and dairy foods (27% of energy as protein, 44% as CHO, and 29% as fat) Standard protein diet low in those foods (16% of energy as protein, 57% as CHO, and 27% as fat) Diets during 12 weeks of energy restriction (6–6.3 MJ/d) and 4 weeks of energy balance (≈8.2 MJ/d)	Calcium excretion, bone markers, dietary analysis	Markers of bone turnover and calcium excretion unchanged between diet groups
Bowen et al., 2004 [3]	Randomised study design Overweight adults were randomly assigned to isoenergetic diets high in dairy protein (M 49.4 ± 3.2 years, F 46.5 ± 2.4 years) or mixed source protein (M 48.7 ± 4.2 years, F 46.1 ± 2.7 years) during 12 weeks of energy restriction and 4 weeks of energy balance (mean ± SEM)	Isoenergetic diets (34% of energy as protein) high in either dairy protein (~2400 mg calcium/d) or mixed protein sources (~500 mg calcium/d)	Calcium excretion, bone markers, dietary analysis	Urinary calcium excretion decreased independently of diet Greater increase in bone resorption marker deoxypyridinoline with mixed protein Increased osteocalcin in mixed protein group
Josse et al., 2012 [94]	Randomised, controlled, parallel intervention design Premenopausal overweight and obese F were randomised into high protein/high dairy (30 ± 1 years), adequate protein/medium dairy (26 ± 1 years) or adequate protein/low dairy protein (28 ± 1 years) (mean ± SEM)	High protein/high dairy: dietary protein (30% of energy), dairy foods (15% energy from protein) and dietary calcium (~1600 mg/d) Adequate protein/medium dairy: dietary protein (15% of energy), dairy foods (7.5% energy from protein) and dietary calcium (~1000 mg/d) Adequate protein/low dairy: dietary protein (15% of energy), dairy foods (<2% energy from protein) and dietary calcium (<500 mg/d)	Bone markers	With low dairy, C-terminal telopeptide of collagen type-I, urinary deoxypyridinoline and osteocalcin increased With high dairy, osteocalcin, amino-terminal propeptide of collagen I increased with resorption markers unchanged

Abbreviations: BMC, bone mineral content; BMD, bone mineral density; CHO, carbohydrate; F, females; M, males; PRAL, potential renal acid load; RET, resistance exercise training; SD, standard deviation; SEM, standard error of the mean.

**Table 3:** Plant-derived proteins: effects on muscle in relation to age, exercise, energy restriction and source.

Reference	Study Design	Protein Composition	Measurements	Key Outcomes
Hartman et al., 2007 [106]	Randomised, controlled, parallel intervention design Soy protein (*n* = 19) vs. milk (*n* = 18) vs. carbohydrate (CHO) control (*n* = 19) Healthy young males (M) (18–30 years). 12 weeks 5 d/week whole-body resistance exercise training (RET)	Soy protein—17.5 g isoenergetic/nitrogenous milk—17.5 g protein CHO—isoenergetic 2 × supplement, post exercise + 1 h	Fat- and bone-free mass (FBFM), fibre cross (CSA), plasma amino acid (AA) profile	No increased FBFM in soy group Soy protein increased type I fibre CSA after 12 weeks, however milk greatly increase type I + II CSA Soy protein increased post-ingestion plasma leucine and EAA profiles similar to milk Increased plasma insulin immediately after ingestion similar to milk
Tang et al., 2009 [97]	Randomised, controlled, parallel intervention design Soy vs. whey vs. casein protein All groups *n* = 6 Healthy young M (22.8 ± 3.9 years, mean ± standard error of the mean (SEM)) Unilateral leg press and knee extension (4 sets, 10–12 repetition maximum (RM))	Soy protein—22.2 g protein, 1.8 g leucine Whey protein—21.4 g protein, 2.3 g leucine Casein protein—21.9 protein, 1.8 g leucine All provided ~10 g EAA Protein drink post exercise.	Rest and exercise muscle fractional synthesis rates (FSR), plasma AA profile	Soy and whey protein increased rest muscle FSR above casein Soy protein + exercise muscle FSR increased above casein protein, however a greater increase was seen in whey protein + exercise Soy protein ingestion increase EAA + leucine profiles above casein protein, with whey protein ingestion increasing both to a greater degree
DeNysschen et al., 2009 [105]	Randomised, double-blind, controlled parallel intervention design Soy protein (*n* = 10) vs. whey protein (*n* = 9) vs. CHO placebo (*n* = 9) Overweight males (21–50 years, mean 38 years, body mass index (BMI) 25–30) 12 weeks 3 d/week whole-body RET	Soy protein—25.8 g Whey protein—26.6 g CHO placebo—0.6 g protein Supplement ingested post-resistance exercise (RE), daily	Body composition, strength, fasting blood measures	All groups increased strength pre to post Total cholesterol decreased in all groups No differences between groups for any measures
Wilkinson et al., 2007 [103]	Randomised cross-over intervention design Soy protein vs. milk *n* = 8 Healthy young M (21.6 ± 0.3 years, mean ± SEM) Unilateral standardised leg workout, 80% 1-RM	Soy protein—18.2 g Isoenergetic/nitrogenous milk—18.2 g protein Protein drink post RE	AV balance-based FSR and fractional breakdown rate (FBR), net balance, plasma AA profile	A significant, but lower increase in total AA and muscle FSR after consumption of soy protein vs. milk Soy protein ingestion resulted in a shorter period of positive net protein balance and area under the curve compared to milk Total AA net balance remained elevated after milk consumption vs. soy protein
Luiking et al., 2011 [108]	Randomised, single-blind parallel intervention design Soy protein (*n* = 10) vs. casein protein (*n* = 12) Healthy young adults (M/females (F) 50:50, 22 ± 1 years, mean ± SEM)	Soy protein—3.4 g protein/100 mL Isonitrogenous casein protein—2.95 g/100 mL Enteral ingestion (2 mL/kg/bw/h)	AV balance based FSR & FBR, net balance, plasma AA profile	Greater net uptake of glutamate, serine, histidine and lysine from casein vs. soy protein Reduced intramuscular branch AA concentrations from soy ingestion compared to casein No differences in muscle protein synthesis (MPS) or muscle protein breakdown between protein sources
Joy et al., 2013 [23]	Randomised, double-blind, parallel intervention design Rice protein vs. whey protein isolate All groups *n* = 12 Healthy young males (21.3 ± 1.9 years, mean ± standard deviation (SD)) Periodic whole-body RET	Rice protein—48 g protein, 80 mg/g leucine Isonitrogenous whey protein isolate—48 g protein, 115 mg/g leucine Ingested post exercise 3 d/week Control diet provided	Muscle thickness, body composition, strength measures	Both groups increased lean mass (LM), bicep/quadricep thickness, with no differences between groups
Babault et al., 2015 [107]	Randomised, double-blind, controlled parallel intervention design Pea protein (*n* = 53) vs. whey protein (*n* = 54) vs. placebo (*n* = 54) Healthy young M (21.9 ± 3.7 years, mean ± SD) 6 weeks 3 d/week progressive strength training, elbow flexor/extensor	Pea protein—26.6 g protein, 2.9 g leucine Whey protein—23.9 g protein Placebo—3.9 g maltodextrin Ingested twice daily morning/afternoon (post exercise) for 6 weeks	Bicep thickness, maximal voluntary torque, 1-RM	All groups increased bicep thickness compared to baseline after 42 and 82 days, no difference between groups Baseline weakest volunteers supplemented with pea protein demonstrated increased bicep thickness between 42 and 84 days
Candow et al., 2006 [104]	Randomised, double-blind, controlled parallel intervention design Soy protein vs. whey protein vs. placebo All groups *n* = 9 Healthy young adults (M/F 1:2, 23 ± 6 years, mean ± SD) 6 weeks 3 d/week whole-body RET	Soy and whey protein—1.2 g/kg Placebo—1.2 g/kg maltodextrin, isocaloric Ingestion split between 3 equal daily doses pre/post-training and evening	Body composition, strength measures, muscle FBR	Both soy and whey protein groups increased LM and strength greater than the placebo group All groups increased muscle FBR similarly
Yang et al., 2012 [71]	Parallel intervention, controlled design Soy protein 20 g or 40 g vs. whey protein 20 g or 40 g vs. water All groups *n* = 10 Healthy older M (71 ± Unilateral knee extension (3 sets, 10-RM).	Soy protein—20 g protein, 1.6 g leucine Soy protein—40 g protein, 3.2 g leucine Whey protein—20 g protein, 2 g leucine Whey protein—40 g, 4 g leucine Water control Protein drink post exercise	Myofibrillar FSR (rest and RE) plasma AA profile, leucine oxidation	No increase in rest myofibrillar FSR in either 20 or 40 g soy protein groups Increased RE myofibrillar FSR in 40 g soy protein group Significant increases in myofibrillar FSR for all whey protein groups, rest + RE 20 and 40 g soy protein increased leucine oxidation to similar degrees
Deibert et al., 2011 [109]	Randomised controlled intervention design Whole-body RET with/without soy protein Healthy moderately overweight older M (55.7 ± 4.6 years, BMI 27.7 ± 2.1, mean ± SD) 12 weeks 2 d/week progressive whole-body RET	50 g soy protein yoghurt—26.7 g protein Control—RET only Consumed after evening training	Skinfold measures, BMI, strength measures, blood biomarkers	Decreased waist circumference and fat mass and increased fat free mass in soy protein supplemented group Improved glycaemic control and metabolic markers in soy protein-supplemented group Both groups increased in strength and coordination
Gorissen et al., 2016 [99]	Randomised, double-blind, controlled parallel intervention design 35 g wheat protein vs. 35 g or 60 g wheat protein hydrolysate vs. 35 g micellar casein protein, 35 g whey protein All groups *n* = 12 Healthy older M (71 ± 1 years, mean ± SEM) Single protein drink ingestion	Wheat protein—35 g Wheat hydrolysate protein—35 g Wheat hydrolysate protein—60 g Micellar casein protein—35 g Whey protein—35 g Single ingestion	Myofibrillar FSR, plasma AA profile	Ingestion of 35 g wheat protein did not increase myofibrillar FSR as much as 35 g whey or 35 g casein protein 60 g wheat hydrolysate stimulated myofibrillar FSR to a greater degree than 35 g whey protein 2–4 h post-ingestion Whey protein ingestion had a greater plasma leucine increase compared to 60 g wheat hydrolysate protein Plasma AA content was more persistent following 60 g wheat hydrolysate ingestion
Oikawa et al., 2020 [102]	Single blind, parallel group design 24 F randomised to potato protein (*n* = 12, 20 ± 3) or control (*n* = 12, 21 ± 3) diet for 2 weeks plus unilateral RET (3 ×/weeks) (mean ± SD)	Potato protein—25 g 2 ×/d (1.6 g/kg/d total protein) Control—0.8 g/kg/d total protein (breakdown of AA composition within each supplement can be found in original article)	Myofibrillar protein synthesis, cell signalling, baseline body composition and strength, dietary analysis	No difference in total kcals or percentage fat intake between groups Protein intake was significantly greater in the potato protein group compared to control MPS increased above baseline at rest in the potato protein, but not control, group MPS increased similarly above baseline with exercise in both groups In response to exercise, total protein kinase B (PKB/Akt) increased compared to baseline Main effect of time for total mechanistic target of rapamycin and ribosomal protein s6

Abbreviations: AA, amino acids; BMI, body mass index; CHO, carbohydrate; CSA, cross-sectional area; EAA, essential amino acid, FBFM, fat- and bone-free mass; F, females; FBR; fractional breakdown rate; FSR, fractional synthesis rate; LM, lean mass; M, males; MPS, muscle protein synthesis; RE, resistance exercise; RET, resistance exercise training; RM, repetition maximum; SD, standard deviation; SEM, standard error of the mean.

**Table 4:** Plant-derived proteins: effects on bone in relation to age, exercise, energy restriction and source.

Reference	Study Design	Protein Composition	Measurements	Key Outcomes
Roughead et al., 2005 [87]	Randomised cross-over intervention study design Low meat soy supplemented vs. high meat *n* = 13 7 weeks, healthy postmenopausal females (F) (59.9 ± 5 years, mean ± standard deviation (SD))	Low meat soy supplemented—55 g/d meat, 25 g soy protein High meat—170 g/g meat All meals provided	Calcium retention, urine composition, blood biomarkers of bone mineral status	No difference in calcium retention between groups No change in blood biomarkers of bone mineral status (i.e., 25-OH vitamin D, parathyroid hormone, insulin-like growth factor-I.
Kreijkamp-Kaspers et al., 2004 [119]	Randomised, double-blind, controlled parallel intervention design Soy protein supplement (*n* = 88) vs. milk protein supplement (*n* = 87) 12 months, healthy postmenopausal F (66 ± 5 years)	Soy protein—25.6 g protein Milk protein—25.6 g protein Single daily ingestion	Hip and lumbar spine bone mineral density (BMD), plasma lipid profiles	No difference in BMD from supplementation No change in plasma lipid profiles
Alekel et al., 2000 [116]	Randomised, double-blind, controlled parallel intervention design Isoflavone-rich soy protein (*n* = 24) vs. isoflavone-poor soy protein (*n* = 24) vs. whey protein control (*n* = 21) 24 weeks supplementation postmenopausal F (42–62 years, mean 50 years)	All groups 40 g protein/d, 160 mg calcium/d Isoflavone-rich 80.4 mg aglycone Isoflavone-poor 4.4 g aglycone Single daily 500 kcal muffin (20 g protein) as a meal replacement	Lumbar spine BMD and bone mineral content (BMC)	Both soy protein groups did not significantly decrease BMD, whereas the whey control group did BMC increase in the isoflavone-rich soy group and decreased in the whey protein control group.
Liu et al., 2010 [120]	Randomised, double-blind, controlled parallel intervention design Soy protein + isoflavone whey protein + isoflavone whey protein control 6 months postmenopausal F (56.1 ± 4.3 years, mean ± SD)	Soy protein—15 g, 100 mg isoflavone Whey protein—15 g, 100 mg isoflavone Whey protein—15 g Daily supplementation	Body composition	Soy protein with isoflavone supplementation demonstrated small but significant improvements in body weight, body mass index and body fat percentage

Abbreviations: BMC, bone mineral content; BMD, bone mineral density; F, females; SD, standard deviation.

**Table 5:** Collagen-derived proteins: effects on muscle in relation to age, exercise, energy restriction and source.

Reference	Study Design	Protein Composition	Measurements	Key Outcomes
Oikawa et al., 2020 [27]	Double-blind, parallel group, randomised controlled trial (RCT) within-subject design (unilateral leg—rest, contralateral leg—resistance exercise (RE)) 22 healthy older female (F) (*n* = 11/group, 69 ± 3 years, mean ± standard deviation (SD)) Randomised to collagen protein or whey protein 2 ×/d for 6 d and unilateral RE twice during 6 d period	Collagen protein—30 g amino acids (AA) of hydrolysed collagen protein Whey protein—30 g AA of whey protein isolate (breakdown of AA composition within each supplement can be found in original article)	Myofibrillar and collagen protein synthesis, cell signalling, baseline body composition and strength	Plasma leucine concentrations increased above baseline post whey protein, but not collagen peptide supplementation Myofibrillar muscle protein synthesis (MPS) increased at rest and post-RE following whey protein, but only increased post-RE following collagen peptide supplementation Collagen peptide supplementation did not influence integrated myofibrillar MPS Rates of integrated myofibrillar MPS significantly greater in whey protein than collagen peptide supplementation
Kirmse et al., 2019 [128] (uses data set from Oertzen-Hagemann et al., 2019)	Randomised, double-blind, placebo-controlled design 57 moderately trained males (M) (24 ± 3 years, mean ± SD) were randomised to full-body resistance exercise training (RET) 3 ×/week for 12 weeks and collagen peptide (*n* = 29) or placebo (*n* = 28) Supplements taken daily for 12 weeks	Hydrolysed collagen peptide—15 g/d Placebo—15 g/d noncaloric silicon dioxide	Body composition, muscle thickness, strength, muscle fibre cross sectional area (CSA), dietary analysis	Strength and type II CSA increased in both groups Fat free mass (FFM) significantly increased in the collagen peptide group, not placebo Body fat mass (FM) did not change in the collagen peptide group but increase in the placebo group No difference in macronutrient intake between groups Protein intake was 1.81 ± 0.42 and 1.74 ± 0.5 g/kg/d in collagen and placebo groups, respectively
Zdzieblik et al., 2015 [26]	Randomised, double-blind, placebo-controlled design 53 older (72.2 ± 4.68 years, mean ± SD) sarcopenic M randomised to full body RET 3 ×/week for 12 weeks and collagen peptide (*n* = 26) or placebo (*n* = 27) Supplements taken daily for 12 weeks	Collagen peptide—15 g/d Placebo—15 g/d silicon dioxide (breakdown of AA composition within collagen peptide supplement can be found in original article)	Body composition, strength, dietary analysis	Increase in FFM and strength greater in collagen peptide versus placebo group Decrease in FM was greater in collagen peptide versus placebo group No difference in dietary intake between groups pre or post intervention and neither were protein deficient
Jendricke et al., 2019 [135]	Randomised, double-blind, placebo-controlled design 77 premenopausal untrained F were randomised to full body RET 3 ×/week for 12 weeks and collagen peptide (*n* = 40, 38.3 ± 8.7 years) or placebo (*n* = 37, 41.6 ± 6.9 years) (mean ± SD) Supplements taken daily for 12 weeks	Collagen peptide—15 g/d Placebo—15 g/d noncaloric silicon dioxide	Body composition, strength	Increase in FFM and hand grip strength was higher in collagen peptide versus placebo group Decrease in percentage body fat was greater in collagen peptide versus placebo group
Oertzen-Hagemann et al., 2019 [28]	Randomised, double-blind, placebo-controlled design 25 M (24.2 ± 2.6 years, mean ± SD) were randomised to full body RET 3 ×/week for 12 weeks and collagen peptide (*n* = 12) or placebo (*n* = 13) Supplements taken daily for 12 weeks	Hydrolysed collagen peptide—15 g/d Placebo—15 g/d noncaloric silicon dioxide	Body composition, strength, proteome	Collagen peptide is bioactive, demonstrated by increased circulating levels of hydroxyproline 2 h following collagen peptide ingestion Body mass and FFM higher in collagen peptide group versus placebo 221 higher abundant proteins identified in collagen peptide group versus on 44 in placebo (proteomic analysis) Upregulated proteins in the collagen peptide group mostly associated with protein metabolism of contractile fibres
Hays et al., 2009 [127]	Double-blind, randomised, cross-over design 9 healthy F (71 ± 1 years, mean ± standard error of the mean (SEM)) completed 2 × 15 d trials (7 d wash-out period in between) Each trial consisted of consuming 0.8 g protein/kg body weight/d with either whey protein or collagen peptide intended to provide ~0.4 g/kg body weight/d	Hydrolysed collagen peptide—~0.4 g/kg body weight/d Whey protein—~0.4 g/kg body weight/d	Body composition, nitrogen balance, dietary analysis	Body weight decreased after whey but not collagen protein intake Nitrogen excretion was higher during whey versus collagen protein intake No difference in macronutrient intake between collagen peptide and whey protein groups (protein intake was 0.82 ± 0.04 g/kg/d)
Oikawa et al., 2018 [77]	Double-blind, parallel group, RCT 16 M (69 ± 3 years) and 15 F (68 ± 4 years) were randomised to collagen peptide (*n* = 15) or whey protein (*n* = 16) and completed 4 phases: 1. 1-week energy balance 2. 1-week energy restriction (−500 kcal/d) and protein supplementation (1.6.g protein/kg/d with 45 ± 9% from whey protein (30 g 2 ×/d) or collagen peptide (30 g 2 ×/d)) 3. 2-week energy restriction with step reduction (≤750 steps/d) 4. 1-week habitual activity (continuing the high protein supplementation protocol) (mean ± SD)	Hydrolysed collagen peptide—30 g Whey protein isolate—30 g (breakdown of AA composition within each supplement can be found in original article)	Myofibrillar MPS, body composition, fascicle CSA, inflammation, insulin sensitivity	Protein supplementation (whey protein or collagen peptide) did not prevent leg LM loss during energy restriction and energy restriction with step reduction Whey protein, but not collagen peptide, augmented lean body mass, leg LM and MPS during habitual activity MPS remained suppressed during the energy restriction with step reduction and habitual activity phases in the collagen peptide group
Impey et al., 2018 [129]	Repeated-measures, counterbalanced design 7–9 d wash-out period 8 recreational M cyclists (25 ± 3 years, mean ± SD) completed an exercise trial in conditions of reduced carbohydrate with hydrolysed collagen or whey protein consumed before, during and after exercise	Hydrolysed collagen blend—22 g (66 g total) taken pre, during and post-exercise Whey protein—22 g (66 g total) taken pre, during and post-exercise	Cell signalling, muscle mitochondria markers	No effect of hydrolysed collagen (or whey protein) on markers of muscle mitochondrial adaptations Hydrolysed collagen supplementation increased anabolic signalling but to a lesser extent than whey protein
Clifford et al., 2019 [29]	Double-blind, placebo-controlled, independent group design 24 recreationally active M were randomised to collagen peptide (*n* = 12, 24.1 ± 4.3 years) or placebo (*n* = 12, 24.8 ± 4.8 years) supplementation 7 d before and 2 d after exercise (mean ± SD)	Collagen peptide—20 g/d Isoenergetic and isovolumic placebo—20 g/d	Muscle function, dietary analysis	Countermovement jump recovered quicker following collagen peptide supplementation (versus placebo) No difference in macronutrient intake between groups throughout the study Protein intake was 1.26 ± 0.46 and 1.18 ± 0.27 g/kg/bm^−1^ for collagen peptide and placebo groups, respectively.
Rindom et al., 2016 [132]	Double-blind, randomised, cross-over design 12 young M (24.6 ± 2.1 years, mean ± SD) completed 1 week of intense full-body RET (4 RET sessions) whilst consuming collagen protein or whey protein, followed by 3 weeks recovery, then completed another 1-week period of intense RET whilst consuming collagen or whey protein (opposite to the type ingested during the first week)	Collagen protein—20 g/d Whey protein—20 g/d During the intense RET period, all volunteers received 1.4 g protein/kg bodyweight in addition to the study supplement (i.e., whey/collagen protein)	Muscle function	48 h after the final exercise bout, maximal voluntary contraction had returned to baseline in both groups. No difference was noted between whey or collagen protein groups at any timepoint 48 h after the final exercise bout, counter movement jump (CMJ) height had returned to baseline in the collagen protein, but not whey protein, supplemented group 3 h after the final exercise bout, whey protein supplemented group displayed attenuated losses in CMJ compared to collagen protein
Oikawa et al., 2019 [130]	Double-blind, randomised, cross-over design 4 d wash-out 11 endurance trained adults (M *n* = 5, F *n* = 6, 24 ± 4 years, mean ± SD) engaged in daily high-intensity interval training with hydrolysed collagen or α-lactalbumin supplementation for 3 d	Hydrolysed collagen peptides—60 g/d α-lactalbumin—60 g/d (breakdown of AA composition within each supplement can be found in original article)	Myofibrillar and sarcoplasmic MPS, dietary analysis	Plasma leucine and tryptophan concentrations were greater following α-lactalbumin compared to hydrolysed collagen supplementation Exercise-induced increased in myofibrillar and sarcoplasmic MPS were greater with α-lactalbumin compared to hydrolysed collagen supplementation No differences in macronutrient intake between groups
Centner et al., 2019 [137]	Prospective, randomised, placebo-controlled design Older M randomised to 8 weeks blood flow resistance (BFR) training with collagen hydrolysate (*n* = 11, 61.7 ± 5.5 years) or 8 weeks BFR training with placebo (*n* = 11, 56.6 ± 6.1 years) or no training with collagen hydrolysate (control) (*n* = 8, 62.5 ± 10.5 years) (mean ± SD)	Collagen hydrolysate—15 g/d Placebo—silicon dioxide—15 g/d	CSA and muscle function	Muscle CSA increase in BFR-collagen hydrolysate (+6.7% ± 3.2%) and BFR-placebo (+5.7% ± 2.7%) but not in control 1-repition maximum strength increased in BRF-collagen hydrolysate (+10.2% ± 24.8%), and BFR-placebo (+4.8% ± 11.4%) but not control, relative to pre-study levels

Abbreviations: AA, amino acid; BFR, blood flow restriction; counter movement jump, CMJ; CSA, cross-sectional area; F, females; FFM, fat-free mass; FM, fat mass; M, males; MPS, muscle protein synthesis; RCT, randomised controlled trial; RE, resistance exercise; RET, resistance exercise training; SD, standard deviation; SEM, standard error of the mean.

**Table 6:** Collagen-derived proteins: effects on bone in relation to age, exercise, energy restriction and source.

Reference	Study Design	Protein Composition	Measurements	Key Outcomes
Zdzieblik et al., 2015 [26] (also studied effects on muscle, see Table 5)	Randomised, double-blind, placebo-controlled design 53 older (72.2 ± 4.68 years, mean ± standard deviation (SD)) sarcopenic males (M) randomised to full body resistance exercise training (RET) 3 ×/week for 12 weeks and collagen peptide (*n* = 26) or placebo (*n* = 27) Supplements taken daily for 12 weeks	Collagen peptide—15 g/d Placebo—15 g/d silicon dioxide (breakdown of amino acid composition within collagen peptide supplement can be found in original article)	Bone mass, dietary analysis	No potentiating effect of collagen peptide on bone mass (beyond that of RET alone) No difference in dietary intake between groups pre- or post-intervention and neither were protein deficient
Clifford et al., 2019 [29] (also studied effects on muscle, see table above)	Double-blind, placebo-controlled, independent group design 24 recreationally active M were randomised to collagen peptide (*n* = 12, 24.1 ± 4.3 years) or placebo (*n* = 2, 24.8 ± 4.8 years) supplementation 7 d before and 2 d after exercise (mean ± SD)	Collagen peptide—20 g/d Isoenergetic and isovolumic placebo—20 g/d	Bone turnover markers, dietary analysis	Collagen peptide had no effect on markers of bone turnover No difference in macronutrient intake between groups throughout the study No difference in macronutrient intake between collagen peptide and placebo groups. Protein intake was 1.26 ± 0.46 and 1.18 ± 0.27 g/kg/bm^−1^ for collagen peptide and placebo groups, respectively
König et al., 2018 [141]	Randomised, double-blind, placebo-controlled design Postmenopausal females (F) randomised to collagen peptide (*n* = 66, 63.8 ± 7.4 years) or placebo (*n* = 65, 64.9 ± 7.1 years) supplementation for 12 months (mean ± SD)	Collagen peptide—5 g/d Maltodextrin (placebo)—5 g/d	Bone mineral density (BMD), bone turnover markers, dietary analysis	BMD increased following collagen peptide supplementation (no change in placebo) Amino-terminal propeptide of collagen I (P1NP) increased following collagen peptide supplementation C-terminal telopeptide of collagen type-I increased following placebo supplementation No difference in macro or micronutrient intake between the groups pre or post intervention and neither were protein deficient
Shaw et al., 2017 [126]	Double-blind, randomised, cross-over design 4 d washout period 8 healthy recreationally active M (27 ± 6 years, mean ± standard error of the mean) provided placebo, 5 or 15 g vitamin-C-enriched gelatin and completed rope skipping exercise, 3 ×/d for 3 days	5 g vitamin-C (48 mg)-enriched gelatin 15 g vitamin-C (48 mg)-enriched gelatin Maltodextrin (placebo)—weight and calorie matched	Bone turnover marker	15 g vitamin-C-enriched gelatin increased circulating P1NP more so than placebo and 5 g
Elam et al., 2014 [140]	Randomised, double-blind, placebo-controlled design 39 postmenopausal F (55.7 ± 3.3 years, mean ± SD) randomised to daily hydrolysed calcium-collagen chelate or placebo supplementation for 12 months	Hydrolysed calcium-collagen chelate—5 g/d (with 500 mg elemental calcium, 200 IU vitamin D_3_) Control—500 mg elemental calcium, 200 IU vitamin D_3_	Total body, lumbar and hip BMD, bone turnover markers	Loss of total body BMD was lower following 12 months supplementation of hydrolysed calcium-collagen chelate versus control (*n* = 22 at 12 months follow up) Sclerostin and tartrate-resistant acid phosphatase isoform 5b (TRAP5b) were lower and bone-specific alkaline phosphatase/TRAP5b ratio was higher following 6 months supplementation of hydrolysed calcium-collagen chelate versus control

Abbreviations: BMD, bone mineral density; F, females; M, males; P1NP, amino-terminal propeptide of collagen I; RET, resistance exercise training; SD, standard deviation; TRAP5b, tartrate-resistant acid phosphatase isoform 5b.
